# Structural basis of the anti-ageing effects of polyphenolics: mitigation of oxidative stress

**DOI:** 10.1186/s13065-020-00696-0

**Published:** 2020-08-10

**Authors:** Adam Rolt, Lynne S. Cox

**Affiliations:** Department of Biochemistry, University of Oxford, South Parks Road, Oxford, OX1 3QU UK

**Keywords:** Ageing, Aging, Oxidative stress, Inflammation, Antioxidants, Polyphenols, Stilbenoids, Flavonoids, Chalcones, NRF2

## Abstract

Ageing, and particularly the onset of age-related diseases, is associated with tissue dysfunction and macromolecular damage, some of which can be attributed to accumulation of oxidative damage. Polyphenolic natural products such as stilbenoids, flavonoids and chalcones have been shown to be effective at ameliorating several age-related phenotypes, including oxidative stress, inflammation, impaired proteostasis and cellular senescence, both in vitro and in vivo. Here we aim to identify the structural basis underlying the pharmacology of polyphenols towards ROS and related biochemical pathways involved in age-related disease. We compile and describe SAR trends across different polyphenol chemotypes including stilbenoids, flavonoids and chalcones, review their different molecular targets and indications, and identify common structural ground between chemotypes and mechanisms of action. In particular, we focus on the structural requirements for the direct scavenging of reactive oxygen/nitrogen species such as radicals as well as coordination of a broader antioxidant response. We further suggest that it is important to consider multiple (rather than single) biological activities when identifying and developing new medicinal chemistry entities with utility in modulating complex biological properties such as cell ageing.

## Background

### Biological ageing and the potential of polyphenols as anti-ageing therapeutics

Major demographic and clinical challenges arise from the rapid increase in the number of people aged over 65 years; such individuals are at risk of developing multiple age-related diseases [1]. The free radical theory of ageing, mooted over 60 years ago [2], suggests that reactive oxygen species (ROS), generated metabolically within eukaryotic cells or from exogenous sources [3], cause macromolecular damage which is detrimental to health. While the importance of free radicals to ageing phenotypes has been widely questioned [4], the urgent need to identify new drug-like molecules that can modulate the causes of age-related diseases [5, 6] has led to a resurgence of interest in anti-oxidants, particularly natural products and their derivatives with polypharmacological properties.

Polyphenolic organic compounds were originally identified as natural products synthesized by plants to serve as pigments, anti-microbial agents (phytoalexins), and in signalling changes in plant state (e.g. growth, ripening) [7]. Their presence in foods associated with human health and longevity benefits, such as fruits and vegetables, tea, cocoa beans and olives, is suggestive that they may play an important dietary role in humans [8]. Understanding the mechanistic basis for health benefits, particularly in prevention and/or treatment of inflammatory and/or age-related diseases, i.e. how disparate molecular structures lead to overlapping phenotypic impacts, requires systematic analysis of classes of polyphenols so that structure–activity relationships (SAR) can be made clear.

Polyphenolic natural products such as stilbenoids, flavonoids and chalcones possess a varied and interesting pharmacological profile marked by interactions with a broad range of biological targets, as well as target-decoupled structurally-derived pharmacology [9–12]. Polyphenolic compounds have been shown to modulate the redox status of cells, to alter cellular signalling and to help prevent the accumulation of damage in long-lived biological molecules such as lipids, proteins and nucleic acids. This is accomplished both directly, through scavenging of reactive oxygen species, and indirectly, via interaction with transcription factors which coordinate the antioxidant response. Additionally, polyphenols can damp down inflammatory signalling, modulate nutrient sensing pathways, and induce the selective apoptosis of senescent cells. Importantly, these biological processes become dysfunctional with age and are causative in the pathogenesis of age-related disease [5, 6, 13, 14]. Hence there is renewed interest in developing polyphenol scaffolds and chemotypes as new therapeutics to ameliorate age-related disease.

Deciphering the precise molecular mechanisms of action of polyphenols in altering biological phenotypes of age-related disease is challenging due to the complexity of biological systems, where multiple different biochemical pathways can all contribute to a particular phenotypic outcome such as ageing. Here we aim to identify the structural basis underlying the pharmacology of polyphenols towards oxidative stress and related biochemical pathways involved in age-related disease. We compile and describe structure-activity relationship (SAR) trends across different polyphenol chemotypes including stilbenoids, flavonoids and chalcones, review their different molecular targets and indications, and identify common structural ground between chemotypes and mechanisms of action. In particular, we focus on the structural requirements for the direct scavenging of reactive oxygen/nitrogen species such as radicals as well as coordination of a broader antioxidant response.

## Main text

### Polyphenols have direct anti-oxidant activity by reacting with ROS to form stabilised radicals

Reactive oxygen species (ROS) such as hydroxyl/peroxyl radicals and peroxides are produced through the normal processes of metabolism, e.g. oxidative phosphorylation and ATP generation. Disrupted ROS homeostasis, which is observed with increasing biological age [3], can result either from increased ROS production or diminished ability to remove ROS. Both the source and nature of the reactive species are important in determining biological targets and the nature of ROS-mediated modifications. Notably, different ROS vary greatly in diffusion distances and reactivity [15]. While ROS can act as important signalling molecules [16, 17], they can also irreversibly damage critical macromolecules, including DNA [18]. Furthermore, ROS can induce cellular senescence both in vitro and in vivo through the p53-mediated DNA damage response [19, 20]. Accordingly, sequestration of ROS has been demonstrated to improve health and even extend healthy lifespan in several model organisms [21–26].

**Fig. 1:**
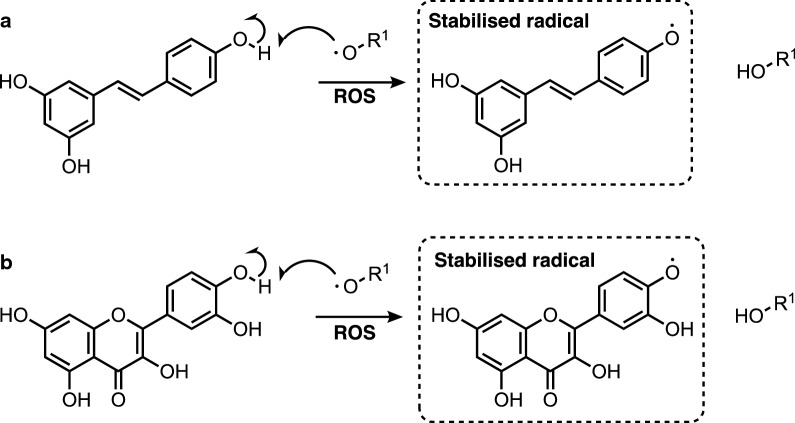
Direct anti-oxidant activity of polyphenols: hydrogen abstraction, quenching of ROS and formation of stabilised stilbenoid (**a**) and flavonoid (**b**) radicals

Polyphenols can react with ROS in a stoichiometric manner to form stabilized radicals, following abstraction of hydrogen, as outlined in Fig. 1. Stabilisation of radicals through delocalisation over the stilbenoid and flavonoid scaffold increases the residence time and diffusion distance of the radicals, enabling stoichiometric clearance by endogenous antioxidants such as glutathione (GSH). As shown in Fig. 1, direct antioxidant activity thus depends on the presence of hydroxyl groups, which both provide a source of labile hydrogen for abstraction, and increase the stability of the radical formed.

### Key antioxidant features of resveratrol and other stilbenoids

**Fig. 2:**
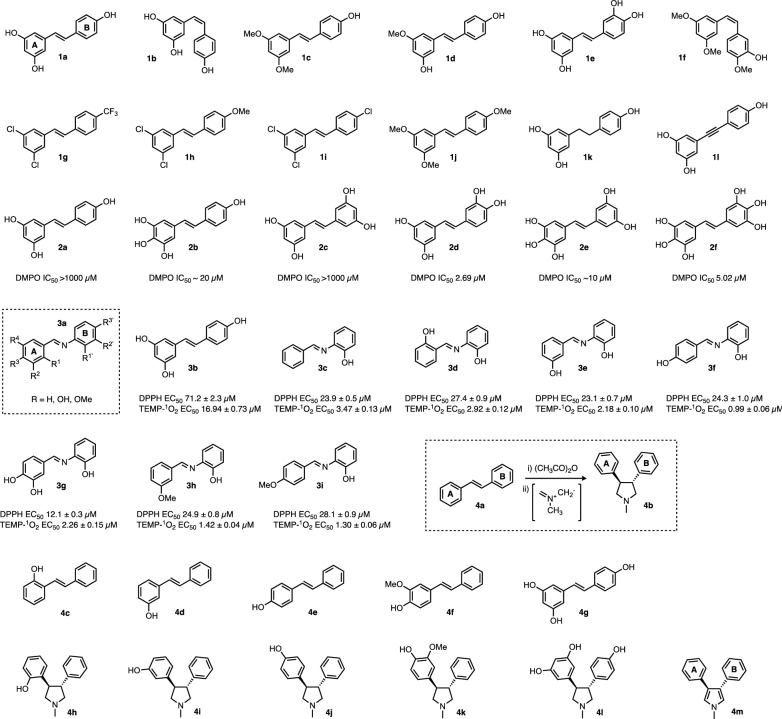
Resveratrol and stilbenoid derivatives. Groups **1–4** reflect the primary research paper(s) in which the series shown was first described (**1a–1l** = ref [25]; **2a–2f** = ref [28]; **3a–3i** = ref [29]; **4a–4m** = Refs. [34, 35]). Where given in the literature, efficacy in antioxidant/radical quenching assays is shown. (Note resveratrol was studied in all four groups and is shown as entries **1a, 2a, 3b** and **4g** for comparison). In vitro activity data—DMPO EC_50_ concentration giving 50% of max effect in DMPO superoxide radical scavenging assay. TEMP-^1^O_2_—singlet oxygen quenching. *DPPH* -DPPH nitrogen radical scavenging assay. General schemes or scaffolds are enclosed in dotted lines

Resveratrol is arguably the most widely studied polyphenol in ageing science [9]. Resveratrol itself (entries **1a, 2a, 3b** and **4g,** Fig. 2), cis-resveratrol **1b** and derivatives **1c–1f** with varying degrees of methylation, all possess direct antioxidative capacity, as determined by an ABTS radical scavenging assay [27]. By contrast, fully substituted derivatives **1g–1j**, lacking critical hydroxyl groups, show greatly diminished or zero antioxidant capabilities [27].

The (E)-olefin functionality in resveratrol is important for potent radical scavenging activity since dihydro-resveratrol **1k**, which lacks the trans-olefin double bond, was shown to have fivefold less potent direct antioxidant activity than resveratrol **1a**, due to diminished ability to stabilise the radical via delocalisation (determined in a DPPH radical scavenging assay) [28, 29]. Furthermore, an alkynyl analogue of resveratrol **1l** was found to be a less potent antioxidant than resveratrol **1a** in a cell based model of oxidative stress [30], indicating requirement for the trans-olefin in resveratrol for potent antioxidant activity.

Given the critical role of a hydroxyl moiety in the formation of a stable stilbenoid radical (Fig. 1), it is therefore unsurprising that increasing the number of hydroxyl groups can increase the rate of radical scavenging. This could be attributed to the presence of ortho-OH groups, which also permit the scavenging of superoxide (O_2_^−·^) radicals. In a series of six stilbenoids (Fig. 2**2a–2f)**, increasing in OH substitution from the tri-hydroxylated resveratrol (entry **2a**) up through to 3,3′,4,4′,5,5′-hexahydroxystilbene, **2f** [31], it was demonstrated that the tetra substituted derivative **2c** possessed inferior efficacy in a kinetic scavenging assay with DMPO radicals compared with resveratrol **2a**. However entries **2b** and **2d** showed greatly improved activity, probably due to the relative substitution patterns, i.e. effect of neighbouring ortho-OH groups, with second order rate constants for DPPH radical trapping in the order of > 10^1^ to 10^3^ -fold higher than resveratrol **2a**. Notably, compounds containing ortho-OH groups **2b, 2d–2f** were also able to scavenge superoxide (O_2_^−·^) radicals with low micromolar efficacy, whereas compounds lacking this ortho-OH functionality did not effectively scavenge O_2_^−·^. While possessing potent antioxidant activity in vitro, **2b** and **2d–2f** were also found to be approximately fourfold more toxic than resveratrol (**2a**) in human cells in culture (toxicity in HL60 cells 18–25 µM EC_90_ for **2b, 2d** and **2e** compared to > 100 µM for resveratrol, **2a**) [31], which could be due to the in situ formation of electrophilic quinone metabolites, as a consequence of increased radical scavenging.

The importance of the trans-olefin bond for resveratrol’s antioxidant activity has been additionally investigated by isosteric replacement of the C=C double bond in resveratrol for an imine, permitting the rapid synthesis of imine resveratrol analogues (IRAs), and evaluation of their biological activity (**3a**, Fig. 2). Twenty-five hydroxylated and methoxylated IRAs [32] were analysed for their ability to quench DPPH radicals and singlet oxygen. Compounds containing a hydroxyl group in the ‘**B**’ ring possessed DPPH scavenging capacity (EC_50_ < 1000 µM), whereas derivatives with a hydroxyl group only present in ring ‘**A**’ did not (EC_50_ > 1000 µM). Structures **3c–3i,** all possessing an ortho-hydroxyl group, were the most potent derivatives in terms of anti-oxidant activity in the DPPH radical scavenging assay (EC_50_ 10–30 µM) [32]; the proximity of the imine nitrogen may permit stabilisation of the electron-deficient oxygen radical through donation [33]. Compound **3g,** with dihydroxyl groups in ring ‘**A**’ as well as ortho-OH in ring ‘**B**’, was the most potent of the IRAs prepared [34]. Additionally, derivatives in the IRA series were more able to quench singlet oxygen than the parent resveratrol **3b** (determined via TEMP-^1^O_2_ adduct formation assay). Imine resveratrol analogues have been investigated in several other independent studies and have shown similar activities and SAR trends [35]. Furthermore the methoxylated IRA **3i** demonstrated anti-inflammatory activity in an in vivo mouse model [36]. These data demonstrate that either olefins or imines in the stilbenoid scaffold can support antioxidant activity, and further emphasise the importance of hydroxyl groups in radical formation and stabilisation.

Extensive structural modifications to the stilbenoid scaffold are permitted whilst still retaining or even enhancing antioxidant activity, provided that the key structural features required for activity are retained. Several tetrahydropyrroyl- (THP) derivatives (**4b**) were analysed alongside their stilbenoids counterparts (**4a**) for their ability to block DNA oxidation in a thiobarbituric acid assay [37, 38]. All THP derivatives were found to be more potent antioxidants than their stilbenoid counterparts. With regards to substituent effects, 2-hydroxy phenyl **4h** was the most potent antioxidant, followed by para **4j** then meta **4i**. Derivatives containing electron donating groups such as 3-methoxy **4k** possessed stronger antioxidant capacity, potentially permitting stabilisation of the radical intermediates. The most potent antioxidant was synthesized through tetrahydropyrrollidisation of resveratrol **4g** itself, to yield **4l**, though no other tri-hydroxylated THP derivatives were studied. The function of the THP group as a chemical bystander was also investigated; it was found that oxidation of the tetrahydropyrrole to the pyrrole **4m** occurs in situ, indicating that the THP group itself is stoichiometrically antioxidant, and increases activity in a mechanistic manner as opposed to just altering the stability of the phenoxy radicals.

### The structural basis of direct antioxidant activity of flavonoids

**Fig. 3:**
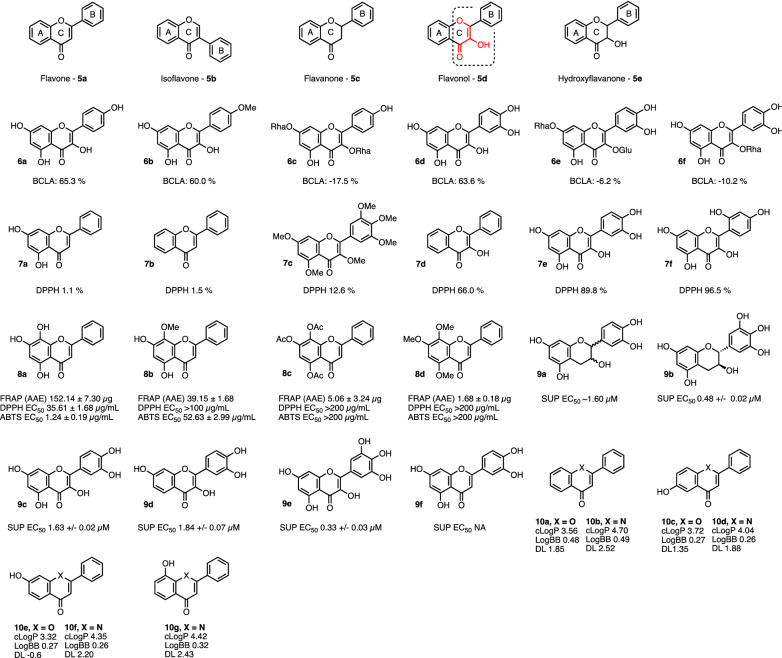
Various flavonoid derivatives and their efficacy in antioxidant/radical quenching assays. The C^2^=C^3^ double bond in conjunction with 3-OH in flavonol (entry **5d**), that confer significant antioxidant properties, are highlighted in red with dotted outline. *In vitro* assay outcomes in which these chemical series are described are given below where these have been reported in the primary papers (see text for details). *BCLA* β-carotene/linoleic acid assay, *FRAP (AAE)* ferric reducing/antioxidant power assay, *DPPH* 2,2-diphenyl-1-picrylhydrazyl, *ABTS* 2,2′-azino-bis-3-ethylbenzothiazoline-6-sulfonic acid, *SUP* superoxide

The antioxidant capabilities of flavonoids follow similar trends to the stilbenoids but are more nuanced, due to the different effects of flavonoid core scaffolds, as illustrated in Fig. 3 entries **5a–5e**.

The HOMO/LUMO energies and ionisation potentials of 10 flavonoid scaffolds including (but not limited to) **5a–5e** were analysed in a density functional theory study, in order to determine the effects of the core scaffold on the theoretical anti-oxidant capacity [39]. A hydroxyl group in the 3-position of the flavonoid scaffold and a C^2^=C^3^ double bond **5d** were found to have higher ionisation potential (IP) (i.e. donate electrons more efficiently), and to stabilise the resultant flavonoid cation free radical (stabilisation energy ∆E_iso_) than scaffolds lacking these structural features. Additionally, ether groups in the scaffold help to stabilise the cation free radical through resonance. The flavonol scaffold **5d** possesses all of these faculties and indeed is experimentally found to be one of the most potent antioxidant structures in the flavonoid class; stepwise masking of these features serves to diminish activity.

The radical scavenging and anti-oxidant capabilities of forty-one flavonoids were benchmarked against each other in DPPH and β-carotene/linoleic acid assays (BCLA—expressed as % antioxidant activity) [40]. The scaffold, **5d**, Fig. 3 was sufficient in itself to provide increased protection against oxidation in the β-carotene/linoleic acid assay. Masking of the C^3^-OH in ring ‘**C**’ **6c, 6e–6f** was in turn sufficient to ablate antioxidant activity. A comparison of radical scavenging in the DPPH assay (expressed as  % radical quenching) demonstrated that compounds with flavonols possessing 4′OH and/or ortho di-OH groups in the B ring were in general the most effective, and activity decreased as these structural features were removed, though there are exceptions, cf. **7a–7f**. As observed in the stilbenoids, the presence of OH groups were required for radical scavenging. While the presence of OH groups in the ring ‘**A**’ was not sufficient for radical scavenging (demonstrated by compounds **7a, 7d, 7e**), catechol functionality (ortho di-OH) in ring ‘**A**’ is sufficient, thus norwogonin **8a** possesses activity in multiple antioxidant assays, but it rapidly loses activity upon partial or complete masking of the hydroxyl groups, as shown in derivatives **8b–8d** [41].

Catechol functional groups are sufficient to permit the scavenging of superoxide radicals (O_2_^−·^) in flavonols/flavanols as well as stilbenoids, as demonstrated by flavonoids **9a–9e,** which also possess the ability to scavenge superoxide radicals [42]. Additionally, comparisons can be drawn between quercetin **9c** and the flavone luteolin **9f**, which is identical to quercetin in terms of hydroxyl group distribution on the phenyl rings ‘**A**’ and ‘**B**’, yet which cannot directly scavenge O_2_^−·^, indicating a greater ability of the flavonol scaffold to stabilise the resultant free radicals (though luteolin does possess indirect antioxidant capacity through the inhibition of xanthine oxidase). Importantly, quercetin (in combination with kinase inhibitor dasatinib) is proving of clinical value in diseases such as idiopathic lung fibrosis and diabetic kidney disease [43, 44]. While benefit has been attributed to senolytic activity (selective killing of senescent cells), antioxidant activity of quercetin in this context may also be important, possibly by preventing further ROS-induced senescence. For both the stilbenoid and flavonoid scaffolds, there are several instances where compounds containing catechol (ortho-dihydroxyl) functionality possess more potent radical scavenging activity than would otherwise be expected from two non-contiguous hydroxyl group based on the SAR, e.g. structures **7a/8a, 9a/9b** etc. This effect is due to neighbouring group participation in hydrogen bonding and in stabilisation of the resultant radical [34, 45].

SAR investigations towards optimisation of radical scavenging ability (i.e. in vitro efficacy) still present only a part of a successful medicinal chemistry campaign—the molecules must also be appropriately absorbed and delivered to their sub-cellular site of action in the body, so improvement of the ADME profile of the molecules is an important consideration in delivering effective therapeutics. In flavonols, the oxygen atom in ring ‘**C**’ can be substituted for nitrogen, as with the respective quinolones (**10a–10g**) [46] which retain their antioxidant capacity, but additionally displaying preferable ADME profiles. While the 7-OH **10f** substitution increased activity in scavenging peroxyl radicals (ORAC), 6-OH and 8-OH quinolones **10d** and **10g** showed increased scavenging activity towards hydroxyl radicals as well as ferrous iron reduction (TBARS and FRAP assays, respectively). Iron levels increase up to 30-fold in pro-inflammatory senescent cells (which also show high endogenous ROS levels) [47], so it will be interesting to test whether iron reduction by quinolones is biologically relevant in age-related diseases where senescence is causative. The quinolones were generally more potent than their flavone counterparts, with the exception of 6-hydroxyflavone vs. 6-hydroxyquinolone (**10d**) in the ORAC assay. Based on electron spin resonance (ESR) analysis of flavonoid antioxidant activities in terms of number and position of OH groups, it has been suggested that rate of reaction rather than stoichiometry may be important for biological protection against ROS [48].

### Polyphenols can upregulate antioxidant response via NRF2

Physiological responses to oxidative stress are co-ordinated within cells by the transcription factor NRF2 (Nuclear factor erythroid 2-related factor 2). Under normoxic conditions, NRF2 levels are low, predominantly due to binding to the negative regulator KEAP1 (Kelch-like ECH-associated protein 1) which facilitates NRF2 ubiquitination and proteasomal degradation [49, 50]. During increased oxidative stress, oxidative cysteine modification of KEAP1 alters its conformation, resulting in diminished binding to NRF2 [51]. NRF2, no longer subject to degradation, translocates to the nucleus where it binds to the antioxidant response element (ARE) upstream of cytoprotective genes, e.g. NAD(P)H quinone oxidoreductase 1 (NQO1), glutathione S-transferase (GST), and glutathione reductase [52, 53], inducing their expression. These factors act to lower ROS and oxidative stress, while simultaneously reducing the cysteines in KEAP1 and subsequently re-establishing a baseline equilibrium of NRF2 activity. Activation of NRF2 signalling, and concomitant amelioration of age-related phenotypes by polyphenols is well documented in vitro and in vivo, and the scaffolds serve as valuable starting points for SAR investigations [54–59]. The stilbenoids resveratrol and pterostilbene specifically have both been demonstrated to upregulate NRF2 activity [55, 60]. A computational study demonstrated that pterostilbene can form stabilising interactions with basic amino acids in the kelch domain of KEAP1 and thus interferes with the breakdown/negative regulation of NRF2 [60].

### NRF2-mediated upregulation of proteasome activity by polyphenols

As well co-ordinating the antioxidant response, activation of NRF2 has been demonstrated to increase proteasomal activity, allowing cells to control protein levels by regulated degradation; while the proteasome degrades proteins tagged with ubiquitin, larger scale protein (and organelle) regulation within cells is mediated by autophagy. The importance of proteostasis is highlighted by the finding of downregulation of autophagy and proteasomal activity in replicatively senescent cells in vitro [61], while inhibition of the proteasome in replicating cells to levels commensurate with that seen in senescence is sufficient to induce premature cellular senescence [62, 63]. Activation of NRF2 using small molecules such as 3*H*-1,2-dithiole-3-thione (D3T, **11**) and 18α-Glycyrrhetinic acid (18α-GA, **12**) increases the expression and activity of the proteasome in a NRF2-dependant manner [64, 65]. Activation of NRF2 by 18α-GA confers resistance against oxidative stress, extends the maximal replicative capacity of human lung HFL-1 fibroblasts, and delays the onset of several phenotypes of cellular senescence [65]. Activation of SKN-1 (worm orthologue of human NRF2) was also demonstrated to upregulate proteasome activity and extend life span in *C. elegans* [66]. Many compounds that upregulate the antioxidant response in vivo contain thiol reactive functionality, for example α, β-unsaturated carbonyl groups, that can undergo conjugate addition reactions to covalently bind to their targets. Examples include chalcones and various flavonoids [67, 68]. Excitingly, the flavonoid 4,4′-dimethoxychalcone (DMC) has recently been reported to have anti-ageing effects across species, potentially through induction of autophagy [69].

NRF2 can also be modulated through imine resveratrol analogues with varied substitution patterns of hydroxylation and methoxylation [70]. Inclusion of an ortho-OH group in the ‘**B**’ ring was found to robustly increase the potency in terms of luciferase expression in an ARE-luciferase reporter assay across several analogues modified across all positions in ring ‘**A**’, c.f. **13b–13f**, but this effect could not be recapitulated by the same ortho-OH substitution in the ‘**A**’ ring. Ortho-OH substitution in the ‘**B**’ ring also increased direct radical scavenging capacity. By combining structural features from promising analogues, two second round derivatives, **13g** and **13h**, were designed which proved to be the most potent derivatives synthesized, increasing ARE-luciferase levels by approximately 10- and 12-fold over control at 15 µM respectively (c.f. resveratrol **13i** leads to a threefold induction at 15 µM). The mechanism of action is proposed to be a covalent inhibition of KEAP1 through nucleophilic addition of a cysteine moiety from KEAP into the electrophilic imine. This interaction could be potentially stabilised through interaction with the OH increasing the reactivity of the incoming thiol, which is illustrated in entry **13j**. The fact that ortho-hydroxyl groups adjacent to electrophilic sites have been shown elsewhere [71, 72] to increase the rate of reactivity with sulfhydryl moieties supports this hypothesis. IRAs represent a promising series since they demonstrate both direct and indirect radical scavenging capacity and are active in vivo [36].

**Fig. 4:**
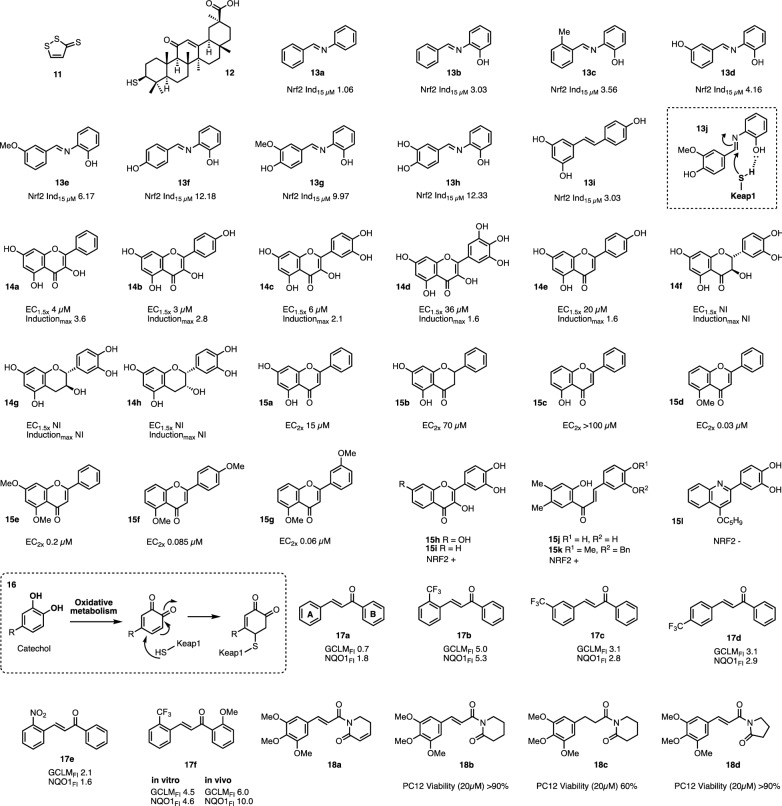
Various polyphenolics involved in investigations of in vitro NRF2 activity. NRF2_Ind15µM_ = fold induction of NRF2 at 15µM, EC_1.5x/2×_ Concentration required to increase activity by 1.5 or 2-fold respectively, *GCLM* glutamate–cysteine ligase modifier subunit, *NQO1* NAD(P)H quinone dehydrogenase 1, *FI* fold induction, *NI* no induction, NRF2 ± refers to positive or negative induction effect on NRF2 (respectively); PC12 is a widely characterised cell line derived from a rat adrenal medulla tumour. Dotted boxes show general schemes of reactions with KEAP1

The structural requirements for upregulation of ARE-driven quinone reductase activity in vitro are highlighted by a comparison between eight flavonoids (Fig. 4, **14a–h**). The α, β-unsaturated ketone in the flavone/flavonol scaffold appears to be required for reactivity, since disruption of this structural feature completely supresses the activity of these compounds. Entries **14a–14e** possess α, β-unsaturated carbonyls with varied distribution of OH groups on rings **A**–**C** and retain activity with micromolar potency. Disruption of the α, β-unsaturated ketone through removing the double bond, ketone, or both (i.e. compounds **14f–14h)** results in loss of activity in terms of NRF2 activation [68].

Measuring ARE-dependent NQO1 expression as a marker for NRF2 transcriptional activity [73], Fahey and colleagues conducted an assessment of a thirty-seven member flavonoid library [74]. Within this set, comparative analysis of the scaffold is possible, since there were several matched molecular pairs, wherein the compounds were identical apart from disruption of the α, β-unsaturated ketone functionality. For example, flavone **15a** was found to be approximately four-fold more potent in supporting NRF2-dependent transcription than its flavanone derivative **15b**. It is of note that of eight flavanones tested, only one possessed comparable potency to its flavone analogue, indicating the importance of intact α, β-unsaturated ketone functionality. 5-methoxy substitution of ring ‘**A**’, **15d–15g,** was associated with potent induction of NQO1, without a concomitant increase in toxicity as assessed in three independent cell lines. The authors note that the differences in activity did not appear to be related to cellular uptake kinetics [74].

The ability to induce NRF2 (and hence indirectly induce an antioxidant response) was removed by altering the flavonol scaffold to the analogous quinolines, hence removing the α, β-unsaturated functionality while maintaining a planar ring system. In a multi-target medicinal chemistry campaign of the flavonol scaffold, several compounds were found to maintain GSH levels following the addition of glutamate [75]. Induction of NRF2 was assessed to determine whether these activities correlated over the series; the parent compound fisetin (entry **15h**) was found to induce NRF2 activity, as well as flavonol **15i** and chalcone derivatives **15j**, **15k**. However, activity is lost upon replacement to the quinoline scaffold (e.g. **15l**), or addition of a basic nitrogen group to ring ‘**B**’, which may target the compound to acidic sub-cellular compartments such as the lysosome, thus sequestering it away from its site of action [76].

Several flavonoid derivatives can still result in inhibition of KEAP1 despite not possessing a Michael acceptor per se. Catechol groups can undergo oxidation under physiological conditions to produce quinones, as depicted in entry **16**. This can be achieved over several steps through various mechanisms, such as oxidation with ROS or redox metalloenzymes, if the cellular environment permits it. The stable quinones can then react with thiols/thiolates on KEAP1, driving an increase in NRF2 activity; alternatively they can be sequestered through glutathione conjugation, which is in turn regenerated [77, 78]. Compounds of this class can increase NRF2 activity in vivo [79]. Furthermore, while some structures do not contain, nor have the propensity to form, α, β-unsaturated carbonyls, ketones themselves (for example in flavanones) are electrophilic and could potentially react with KEAP1 or other sensor-type cysteine proteins forming the respective hemithioacetals; additionally, compounds may also act upon the NRF2/KEAP1 axis in a non-covalent manner [80–85].

NRF2 can similarly be modified by other α, β-unsaturated ketone-containing natural products such as chalcones, from which flavonoids are structurally derived [86]. Notably these compounds exhibit activity while not containing any hydroxyl groups. A library of fifty-nine chalcones (scaffold of entry **17a**) were synthesized and screened (at 100 µM) for their ability to modulate NRF2 signalling, as determined by measuring the fold change in expression of ARE-driven genes GCLM and NQO1 [87]. A large array of methoxy-substituted derivatives at ring ‘**A**’ were analysed in concert with either an ortho, meta, or para-CF_3_ substitution at ring ‘**B**’ (**17b–17d**). Ortho-CF_3_ (**17b**) reliably improved potency of the compounds in terms of fold induction, as compared with meta and para, potentially indicating that a negative inductive effect increases the reactivity of the α, β-unsaturated ketone. Attempts to recapitulate this by substitution of the CF_3_ group for NO_2_ (**17d**) rendered the compounds cytotoxic [87]. The pattern of methoxy substitution on the phenyl ring did not show a consistent or discernible SAR profile: the most potent induction of NRF2 in vitro was seen with the unsubstituted ortho-CF_3_ derivative **17b**, but disubstituted **17f** emerged as the most potent of the series when the compounds were examined in vivo in mouse small intestine following gavage (6- and 10-fold upregulation for GCLM and NQO1 respectively compared to vehicle control) [87].

Piperlongumine (PL, **18a**) is a natural product chalcone-derivative with demonstrated cytoprotective effects against H_2_O_2_ or 6-OHDA-induced oxidative stress; the protective mechanism is probably mediated through NRF2 signalling [88] As with the parent chalcone scaffold, retention of the exocyclic trans double bond was found to be necessary for cytoprotection against oxidative stress, as demonstrated by pairwise analysis of compounds **18b** and **18c**. The olefin present in the lactam ring in parent PL **18a** was not required for potent activation of the antioxidant response. Structures **18b** and **18d** were identified as the most potent derivatives lacking innate cytotoxicity and were shown to induce the nuclear accumulation of NRF2, with a concomitant upregulation of the antioxidant response. Knockdown of NRF2 ablated the cytoprotective effect of these compounds, demonstrating their action through the NRF2 axis [88].

### SAR summary

The ability of both stilbenoids and flavonoids to directly scavenge free radical species is dependent on the presence of a labile hydrogen within the polyphenol, usually from OH, to donate to ROS, and also on the resultant stability of the polyphenol radical once formed. Masking of critical OH groups required for reactivity with ROS ablates potency. Similarly, antioxidant activity of polyphenols is also decreased by destabilisation of the resultant radical through removal of the stilbenoid π-bond (olefin or imine), removing scaffold-defining features of flavonols, or substitution on phenyl groups with substituents that destabilise the radical.

**Fig. 5:**
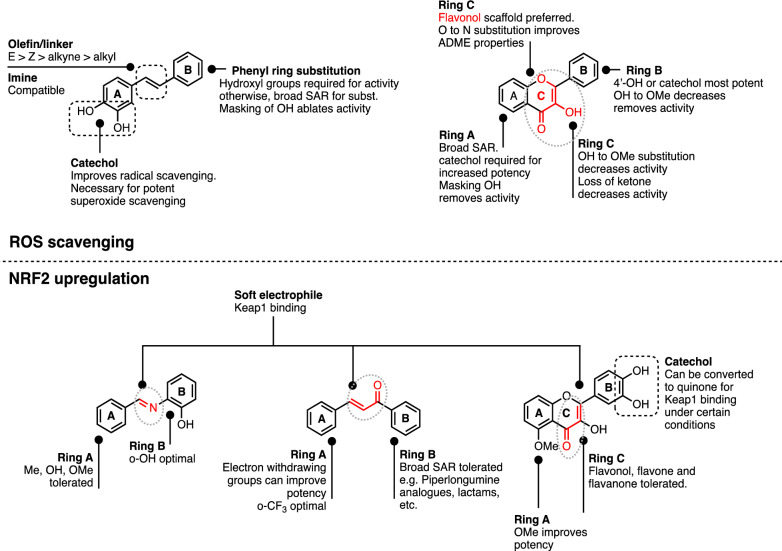
Summary of key features of polyphenols that contribute to direct or indirect antioxidant activity through ROS scavenging or NRF2 upregulation (upper and lower panels respectively). Black dotted boxes indicate catechol and olefin groups; red indicates important groups (also outlined with pale grey dotted ovals)

With regards to NRF2 activation, α, β-unsaturated carbonyl groups appear to be necessary for polyphenols to act as indirect antioxidants though activation of NRF2. Soft electrophiles are preferred since the mechanism is through reaction with KEAP1 cysteine residues (R-SH being a soft nucleophile). To our knowledge, it has not been conclusively demonstrated whether the less reactive α, β-unsaturated carbonyl groups that are present in the scaffold react directly with KEAP1, or whether said scaffold serves to stabilise the formation of more reactive quinone species in the neighbouring phenol ring, which would subsequently react with KEAP1. Regardless of mechanism, the strong dependence on the presence of the α, β-unsaturated carbonyls for efficacy is notable. Flavonoids containing imines and ketones retain some potency. Modifications enhancing electrophilic reactivity appear to also enhance in vitro potency for NRF2 activation. Sensitivity of various structural features towards modification are described in Fig. 5.

## Conclusions

Age-related diseases (ARDs) are complex, systems-dependent phenomena; drugs that act on individual protein targets are therefore unlikely to be effective in disease modification. By contrast, compounds that possess beneficial polypharmacology, i.e. target multiple therapeutic pathways simultaneously, will have a higher likelihood of robustly modulating ARDs. Polyphenolics such as stilbenoids, flavonoids and chalcones are polypharmacologically active in several ARD-relevant biological processes, particularly oxidative stress, inflammation and cellular senescence. The pharmacophores for direct and indirect modulation of oxidative stress, through scavenging of ROS or upregulation of NRF2 activity respectively, are overlapping in some areas and discrete in others, but both direct and indirect activities are likely to be important in the overall molecular mechanisms of these compounds in vivo. Additionally, there are other contributing mechanisms by which specific sub-classes of polyphenolics may affect ROS homeostasis not discussed here, such as inhibition of xanthine oxidase [42]. This complexity mirrors that of the multiple interacting biochemical pathways involved in generating the complex phenotype of biological ageing. In drug design campaigns for anti-ageing therapeutics, we therefore caution against optimising for efficacy in one particular facet of biochemical activity (e.g. in vitro quenching of ROS) without reflecting the complexity of the biological system [89] such as that represented by cell and organismal ageing. Moreover, while transformed cancer cell lines are widely used in drug screening (presumably because of ease of culture and immortality), they are far from ideal as a platform for testing drugs for amelioration of ageing phenotypes; such immortal cells do not age and their biochemical signalling pathways can be highly abnormal. Instead, therefore, we suggest that anti-ageing drug development requires testing in a number of parallel in vitro biochemical assays combined with full phenotypic assays using cell and/or whole organism models directly relevant to ageing, for example replicatively aged primary human cells and short-lived invertebrate organisms with readily scorable ageing outcomes. Such screening programmes are likely to highlight agents with beneficial polypharmacological properties that may prove to have therapeutic benefit in vivo. Polyphenolic natural products therefore represent strong starting points for medicinal chemistry optimisation campaigns in anti-ageing therapeutics.

## Data Availability

All data generated or analysed during this study are included in this published article.

## Abbreviations

6-OHDA: 6-Hydroxydopamine
18α-GA: 18α-Glycyrrhetinic acid
ABTS: 2,2′-Azino-bis-3-ethylbenzothiazoline-6-Sulfonic acid
ARD: Age-related disease
ARE: Antioxidant response element
ATP: Adenosine triphosphate
BCLA: β-CAROTENE/linoleic acid assay
D3T: 3*H*-1,2-Dithiole-3-thione
DMPO: 5,5-Dimethyl-1-pyrroline-*N*-oxide
DNA: Deoxyribonucleic acid
DPPH: 2,2-Diphenyl-1-picrylhydrazyl
ESR: Electron spin resonance
FI: Fold induction
FRAP: Ferric reducing/antioxidant power
FRAP (AAE): Ferric reducing/antioxidant power (ascorbic acid equivalents)
GCLM: Glutamate-cysteine ligase modifier subunit
GSH: Glutathione
GST: Glutathione *S*-transferase
IRA: Imine resveratrol analogues
KEAP1: Kelch-like ECH-associated protein 1
NI: No induction
NRF2: Nuclear factor erythroid 2-related factor 2
ORAC: Oxygen radical absorbance capacity
ROS: Reactive oxygen species
SAR: Structure–activity relationship
SKN-1: Nematode worm orthologue of human NRF2
SUP: Superoxide
TBARS: Thiobarbituric acid reactive substances
TEMP: (2,2,6,6-Tetramethylpiperidin-1-yl)-
THP: Tetrahydropyrroyl
